# Supplementary material for: Doxorubicin Impairs Smooth Muscle Cell Contraction: Novel Insights in Vascular Toxicity
Source: Int J Mol Sci. 2021 Nov 26;22(23):12812. doi: 10.3390/ijms222312812 (PMC8657832; doi:10.3390/ijms222312812)
Supplement: Supplementary file 1 [file ijms-22-12812-s001.zip › ijms-1377305-supplementary.pdf]

International Journal of *Molecular Sciences*

MDPI

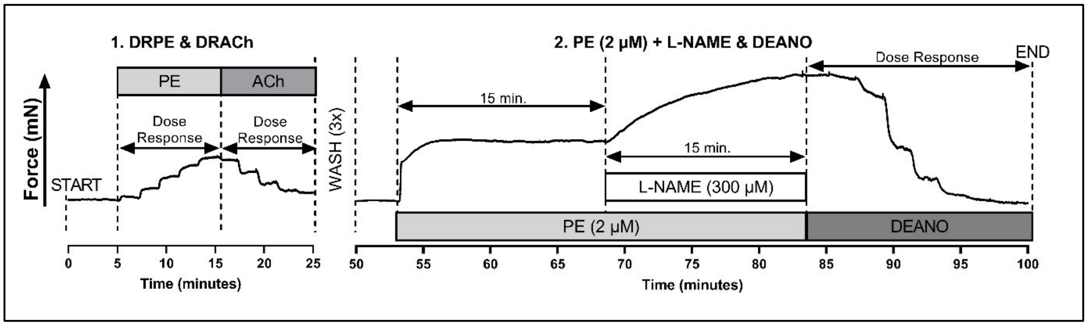

Figure S1: Experimental protocol for vascular reactivity evaluation in aortic segments after *in vivo* DOX treatment.

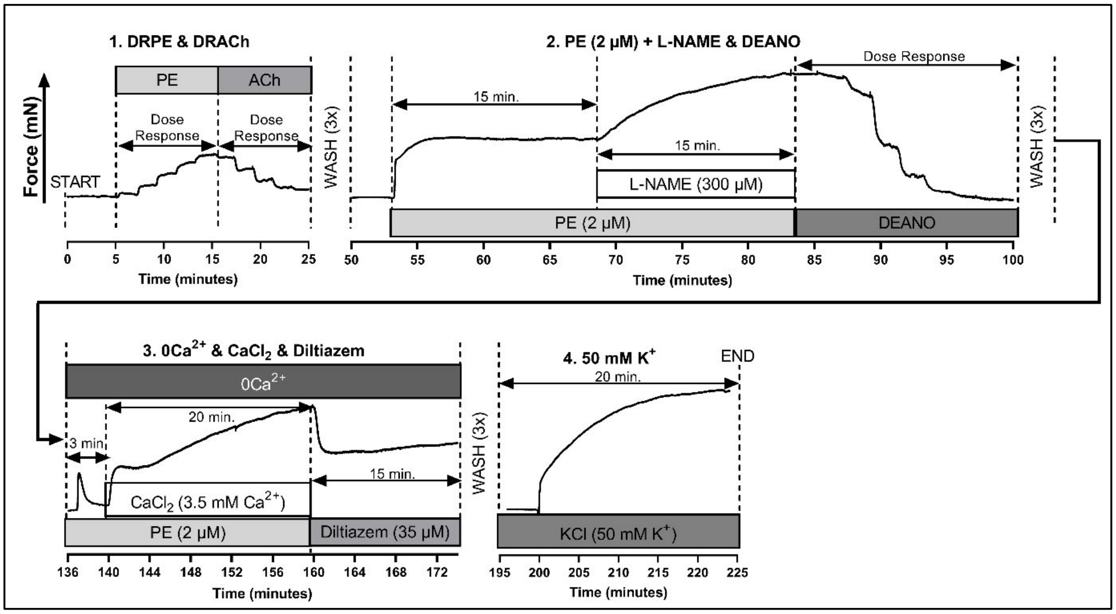

Figure S2: Experimental protocol for vascular reactivity evaluation in aortic segments after *ex vivo* DOX treatment.

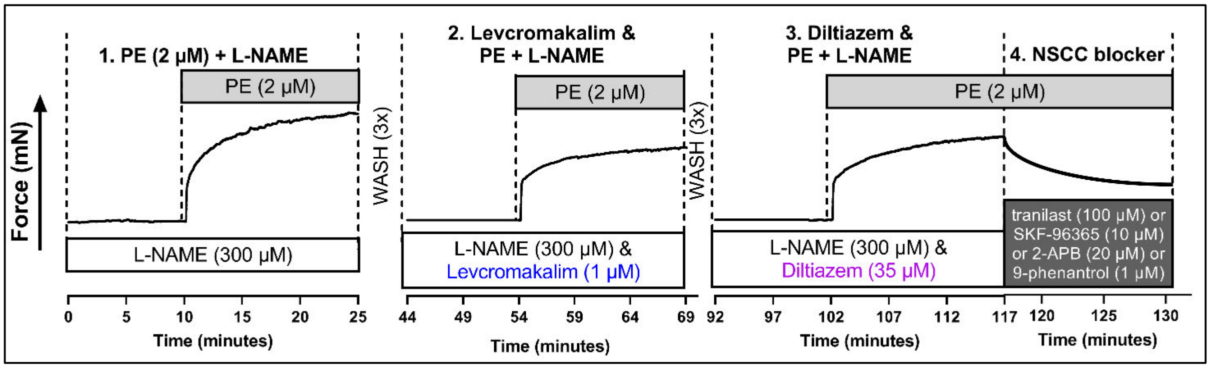

Figure S3: Experimental protocol for investigation of DOX-induced reduction in VSMC contraction after VGCC inhibition.
